# Underreporting of Fatal Congenital Zika Syndrome, Mexico, 2016–2017

**DOI:** 10.3201/eid2508.190106

**Published:** 2019-08

**Authors:** Victor M. Cardenas, Angel Jose Paternina-Caicedo, Ernesto Benito Salvatierra

**Affiliations:** University of Arkansas for Medical Sciences, Little Rock, Arkansas, USA (V.M. Cardenas);; Universidad Nacional de Colombia, Bogota, Colombia (A.J. Paternina-Caicedo);; Unidad San Cristóbal, Chiapas, Mexico (E.B. Salvatierra)

**Keywords:** Zika virus, Zika virus infection, microcephaly, public health surveillance, infant mortality, congenital Zika syndrome, viruses, Mexico

## Abstract

To determine completeness of fatal congenital Zika syndrome reporting in Mexico, we examined data from the Mexican National Institute of Statistics and Geography. We found that an estimated 50% more infants died from microcephaly attributable to congenital Zika syndrome during 2016–2017 than were reported by the existing surveillance system.

Congenital Zika syndrome (CZS), described in Brazil in 2015, consists of a set of congenital malformations (saliently microcephaly) and an increased risk for stillbirth and early childhood death (*1**–**3*). Epidemiologic studies have demonstrated that Zika virus causes CZS (*4*) and that Zika virus–associated birth defects developed in ≈5% of fetuses and newborns of infected pregnant women (*3**,**5**–**7*).

Rates of reported CZS cases in the Americas vary widely. Most (79%) of the 3,720 confirmed cases of CZS reported in the Americas as of January 2018 were reported in Brazil (*8*). The higher reported rates in Brazil could result from the preexisting birth defects registration in Brazil, enhanced by the occurrence of embriopathy associated with use of thalidomide to treat leprosy (*9*). If the 5% prevalence of CZS among neonates of infected pregnant women found in population studies (*3**,**5**–**7*) were applied to the 7,113 pregnant women reported in Mexico as being Zika virus infected (*10*), one would expect ≈355 CZS cases, not the 51 reported as of November 2018 (*11*).

To improve the public health surveillance and research of CZS, we assessed the effects of the Zika virus epidemic on rates of infant death from microcephaly and estimated the completeness of reporting of fatal CZS cases in Mexico. This study was exempt from institutional review board oversight.

## The Study

We accessed tabulated data on infant deaths and births available from the Mexican National Institute of Statistics and Geography for 1998–2017 (*12**,**13*). Using the International Classification of Diseases, 10th Revision, we selected records for infants whose underlying cause of death was coded as microcephaly (Q02X). We used the most recent published report of CZS available from the Mexico Ministry of Health Division of Epidemiology (*13*).

We estimated infant mortality rates by using the number of registered live births per year for the entire country (i.e., cause-specific infant death rates, expressed per 100,000 live births). Because the Zika virus epidemic in Mexico started in November 2015 (*14*), our exposure period of interest was 2016–2017. We identified the baseline period by using joinpoint trend analysis (*15*), a statistical method used to decompose temporal trends (annual percent change [APC]) into meaningful segments. We used the permutation test to identify the most parsimonious results (*15*). We then compared the baseline rate with that of the epidemic period by using the rate ratio and estimating its 95% CI. Infant deaths possibly resulting from the Zika virus epidemic were estimated by using the attributable risk and compared with the number of fatal CZS cases reported by the existing CZS surveillance system. We tested statistical significance by using normal approximation and set the threshold at p = 0.05.

From 1998 through 2017, a total of 467 infants died of microcephaly in Mexico (Table 1). Joinpoint regression identified an overall significant decrease of 6.80% APC (95% CI –11.9% to −1.4%) for 2007–2015 and a statistically significant increase of 27.25% APC for 2016–2017 (95% CI 3.0% to 57.2%) (Figure). On the basis of the results of the trend analysis and the documentation of the first Zika virus outbreak in Mexico during November 2015, we selected the period 2007–2015 as baseline (Table 2).

**Table 1:** Infant deaths from microcephaly and death rates per 100,000 live births, by year, Mexico, 1998–2018

Year	No. infant deaths from microcephaly*	Live births	Rate of infant deaths from microcephaly/100,000 live births
1998	26	2,668,428	0.97
1999	24	2,769,089	0.87
2000	21	2,798,339	0.75
2001	27	2,767,610	0.98
2002	23	2,699,084	0.85
2003	23	2,655,894	0.87
2004	27	2,625,056	1.03
2005	31	2,567,906	1.21
2006	29	2,505,939	1.16
2007	26	2,655,083	0.98
2008	22	2,636,110	0.83
2009	22	2,577,214	0.85
2010	21	2,643,908	0.79
2011	27	2,586,287	1.04
2012	17	2,498,880	0.68
2013	18	2,478,889	0.73
2014	13	2,463,420	0.53
2015	17	2,353,596	0.72
2016	26	2,293,708	1.13
2017	27	2,234,039	1.21

*International Classification of Disease, 10th Revision, code Q02x.

**Figure:**
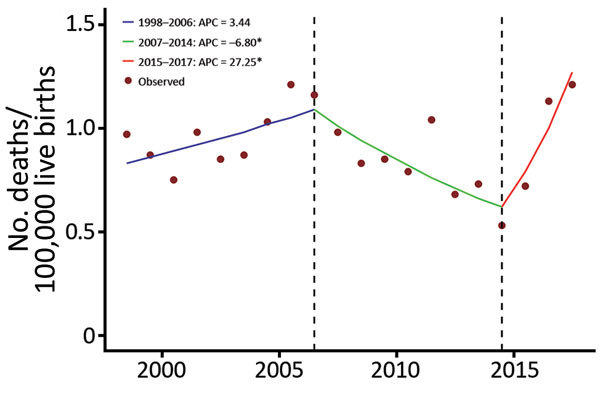
Infant deaths from microcephaly in Mexico, 1998–2017. APC, annual percent change. *p<0.05.

**Table 2:** Infant deaths from microcephaly in Mexico during 2007–2015 and 2016–2017

Period	No. infant deaths from microcephaly*	No. live births	Rate of infant deaths from microcephaly/100,000 live births	Rate ratio (95% CI)
2016–2017	53	4,527,747	1.17	1.5 (1.1–2.0)
2007–2015	183	22,893,387	0.80	Referent

*International Classification of Diseases, 10th Revision, code Q02x.

During the epidemic period (2016–2017), the rate of infant deaths from microcephaly was 1.17 deaths/100,000 live births; during the preceding 4 years (2007–2015), the rate was 0.80 deaths/100,000 live births. Thus, the rate ratio was 1.5 (95% CI 1.1–2.0). The attributable risk was 31.7%.

From January 1, 2016, through November 26, 2018, a total of 51 cases of CZS were reported in Mexico; of these, 11 deaths were reported during 2016–2017. Applying the attributable risk of 31.7% to the 53 reported infant deaths from microcephaly during 2016–2017, we estimated that ≈17 infant deaths from microcephaly were attributable to the Zika virus epidemic. Compared with the 11 reported fatal cases, this estimate resulted in a ratio of 1.5 (95% CI 0.9–2.4), indicating that 50% more infants died of microcephaly caused by CZS than were reported.

## Conclusions

We found evidence that the Zika virus epidemic reversed the declining trend of infant deaths from microcephaly in Mexico and that the number of deaths from microcephaly associated with Zika virus was 50% higher than that reported by the existing CZS surveillance system. In addition, on the basis of the case-fatality rate of 22% for reported CZS, at least 79 cases of CZS would have occurred in 2016–2017. We also observed an increase in the rates of fetal deaths coded as caused by microcephaly in 2016–2017, but we focused our report on infant deaths because the CZS case definition includes only live births.

Our assessment is not without limitations. First, it was limited to fatal CZS and relies on International Classification of Diseases coding. Increased awareness prompted by the Zika epidemic is another potential source of error. Other sources of data such as morbidity (e.g., hospital discharge and other medical records) still need to be evaluated for changes in temporal trends of microcephaly and other manifestations of CZS, such as arthrogryposis, blindness, and deafness. In addition, the accuracy of microcephaly as the underlying cause of death is unknown; microcephaly could have been present among other conditions mentioned in death records but not selected as the underlying cause of death. We believe that death records are prone to underregistration, and yet we found a significant increase in deaths from CZS in the 2 years of the Zika epidemic in Mexico.

Several factors may lead to incomplete reporting of the Zika virus epidemic and CZS. Had primary infection with Zika virus during pregnancy not resulted in CZS, Zika virus would have gone mostly unnoticed, as do many other arboviral infections (e.g., dengue, chikungunya). For instance, the short duration of viremia (3–5 days) complicates confirmatory testing. Although obtaining and testing paired serum specimens would provide more certainty, doing so is logistically harder to achieve. Furthermore, the fact that CZS can occur as a result of Zika virus subclinical infection precludes suspicions and testing.

Reporting of communicable diseases in Mexico, as in other countries, is far from complete. In 1981, we found 2 cases of poliomyelitis for every reported case, and in 1989, we found 1 recorded neonate death from tetanus for every 50 such deaths. However, a good surveillance system does not need to achieve complete reporting to be useful; rather, it should accurately depict the patterns of occurrence of the events or conditions of interest that can lead to their control, presuming existence of effective prevention and control methods.

To improve Zika virus and CZS surveillance in Mexico, resources could be more efficiently used. Zika-endemic areas could be targeted, using active surveillance to monitor the occurrence of microcephaly at birth and flagging neonates born with gestational age and gender-specific head circumference <2 SDs of the reference. The surveillance system could use sentinel sites selected according to the existing risk stratification strategies used for dengue, which could enable extrapolation of the data to the rest of the country. These data would be particularly helpful in *Aedes aegypti* mosquito surveillance and control, which represents an enormous public health challenge.
